# Comparison of molecular subtype distribution in triple-negative inflammatory and non-inflammatory breast cancers

**DOI:** 10.1186/bcr3579

**Published:** 2013-11-25

**Authors:** Hiroko Masuda, Keith A Baggerly, Ying Wang, Takayuki Iwamoto, Takae Brewer, Lajos Pusztai, Kazuharu Kai, Takahiro Kogawa, Pascal Finetti, Daniel Birnbaum, Luc Dirix, Wendy A Woodward, James M Reuben, Savitri Krishnamurthy, W Fraser Symmans, Steven J Van Laere, François Bertucci, Gabriel N Hortobagyi, Naoto T Ueno

**Affiliations:** 1 Morgan Welch Inflammatory Breast Cancer Research Program and Clinic, The University of Texas MD Anderson Cancer Center, Houston, TX, USA; 2 Department of Breast Medical Oncology, The University of Texas MD Anderson Cancer Center, Houston, TX, USA; 3 Department of Bioinformatics and Computer Biology, The University of Texas MD Anderson Cancer Center, Houston, TX, USA; 4 Department of Breast and Endocrine Surgery, Okayama University Hospital, Okayama, Okayama, Japan; 5 Institut Paoli-Calmettes, Marseille, France; 6 Translational Cancer Research Unit-Antwerp, Oncology Center, General Hospital Sint-Augustinus, Wilrijk, Belgium; 7 Laboratory of Gynecological Oncology, Department of Oncology, Catholic University Leuven, Leuven, Belgium; 8 Department of Radiation Oncology, The University of Texas MD Anderson Cancer Center, Houston, TX, USA; 9 Department of Hematopathology, The University of Texas MD Anderson Cancer Center, Houston, TX, USA; 10 Department of Pathology, The University of Texas MD Anderson Cancer Center, Houston, TX, USA; 11 Morgan Welch Inflammatory Breast Cancer Research Program and Clinic, Section of Translational Breast Cancer Research, Department of Breast Medical Oncology, The University of Texas MD Anderson Cancer Center, 1515 Holcombe, Unit 1354, Houston, TX 77030, USA

## Abstract

**Introduction:**

Because of its high rate of metastasis, inflammatory breast cancer (IBC) has a poor prognosis compared with non-inflammatory types of breast cancer (non-IBC). In a recent study, Lehmann and colleagues identified seven subtypes of triple-negative breast cancer (TNBC). We hypothesized that the distribution of TNBC subtypes differs between TN-IBC and TN-non-IBC. We determined the subtypes and compared clinical outcomes by subtype in TN-IBC and TN-non-IBC patients.

**Methods:**

We determined TNBC subtypes in a TNBC cohort from the World IBC Consortium for which IBC status was known (39 cases of TN-IBC; 49 cases of TN-non-IBC). We then determined the associations between TNBC subtypes and IBC status and compared clinical outcomes between TNBC subtypes.

**Results:**

We found the seven subtypes exist in both TN-IBC and TN-non-IBC. We found no association between TNBC subtype and IBC status (*P* = 0.47). TNBC subtype did not predict recurrence-free survival. IBC status was not a significant predictor of recurrence-free or overall survival in the TNBC cohort.

**Conclusions:**

Our data show that, like TN-non-IBC, TN-IBC is a heterogeneous disease. Although clinical characteristics differ significantly between IBC and non-IBC, no unique IBC-specific TNBC subtypes were identified by mRNA gene-expression profiles of the tumor. Studies are needed to identify the subtle molecular or microenvironmental differences that contribute to the differing clinical behaviors between TN-IBC and TN-non-IBC.

## Introduction

Inflammatory breast cancer (IBC), with its clinical and biological characteristics of rapid proliferation, is the most aggressive form of breast cancer [1]. The prevalence of IBC in the United States ranges from 1% to 5% of breast cancer cases [2,3]; however, IBC has a high recurrence/metastasis rate compared with that of non-IBC and commonly leads to breast cancer death. Because current treatments do not result in long-term eradication of disease, there is an urgent need to define the biology of IBC to develop molecularly targeted therapies that may prove more effective.

IBC is diagnosed on the basis of its well-recognized clinical characteristics [4]. Because IBC occurs rarely, its biologic behavior has not been elucidated clearly, and we do not have a clear molecular signature of this disease.

Recently, both *in vivo* and *in vitro* experiments identified genes that contribute to the aggressive phenotype of IBC [5-15]. Bieche *et al*. [5] reported that cell-proliferation genes are more strongly associated with IBC than with non-IBC and that tumoral processes, including signaling pathways associated with inflammation, cell transformation, invasive growth, and angiogenesis, are altered more in IBC than in non-IBC. Nguyen *et al.*[8], by using expression analysis of human cDNA, discovered that, compared with non-IBC, IBC had significantly higher expression of Ki-67 and genes associated with metabolic pathways and lipid signaling. Their findings confirmed that the IBC phenotype is hyperproliferative. Other studies have also investigated the specific biology of IBC. An *in vitro* study revealed that epithelial-mesenchymal transition (EMT) is associated with the migration and invasion of cells from IBC cell lines [16], and much focus has been placed on determining the role of EMT in tumor progression and metastasis in IBC [17]. Zhang *et al*. [16] reported that the epidermal growth factor receptor (EGFR) pathway was involved in tumor growth and metastasis of IBC through EMT and that EGFR expression was an independent and poor prognostic factor in IBC [18,19].

About 20% to 40% of IBC cases are triple-negative breast cancer (TNBC) [20-22], which has a worse prognosis than breast cancers that are positive for ER, PgR, and/or HER2. In contrast, only 15% to 20% of non-IBC cases are TNBC. Many investigators have speculated that the high percentage of TNBC is one of the reasons that IBC has been associated with a more-aggressive clinical course and decreased overall and breast cancer-specific survival [23]. Recently, Lehmann *et al*. [24] reported that TNBC can be classified into seven subtypes on the basis of differential GE and gene ontologies: basal-like 1 (BL1), basal-like 2 (BL2), immunomodulatory (IM), mesenchymal (M), mesenchymal stem-like (MSL), luminal androgen receptor (LAR), and unstable (UNS). We have validated these subtypes in 146 TNBC patients with gene-expression microarrays obtained from June 2000 to March 2010 at our institution [25].

In the current study, we evaluated these seven TNBC subtypes with respect to IBC. Because IBC behavior is more aggressive and is clinically different from that of non-IBC, we hypothesized that the distribution of TNBC subtypes differs between TN-IBC and TN-non-IBC. We initially speculated that the predominant TNBC subtypes in TN-IBC would be BL1 and MSL, based on the biologic characteristics shown by the gene-set enrichment analysis in the Lehmann *et al*. article. BL1 is associated with the enrichment of proliferation genes and increased Ki-67 expression, and MSL is associated with EMT and is consistent with a claudin-low subtype of breast cancer. We tested our hypothesis by applying the subtypes of Lehmann *et al.* subtypes to a World IBC Consortium dataset that included 39 patients with TN-IBC and 49 patients with TN-non-IBC. We also compared clinical outcomes in TN-IBC and TN-non-IBC patients by subtype to investigate whether these subtypes relate to the poor prognosis of TN-IBC.

## Materials and methods

### Patient cohorts and GE data

Three institutions contributed to the World IBC Consortium dataset: The University of Texas MD Anderson Cancer Center, Houston, TX (patients diagnosed with IBC in 2000 through 2005); General Hospital Sint-Augustinus, Antwerp, Wilrijk, Belgium (1996 through 2009); and Institut Paoli-Calmettes, Marseille, France (1998 through 2008). The dataset also includes 252 non-IBC patients. We obtained clinical data and GE profiles, as well as breast cancer samples, for 389 patients from these three institutions. All patients gave written informed consent for voluntary participation, and this study was approved by the institutional review boards of all three participating centers. Samples were stored according to each institution’s criteria, as described in the supplemental information (see Additional file 1). IBC was defined according to the consensus diagnostic criteria published by Dawood *et al*. [22]. In total, 137 IBC patients (including 39 TN-IBC patients) and 252 non-IBC patients (including 49 TN-non-IBC patients) were in the World IBC Consortium database.

All patients were treated with use of a multidisciplinary approach according to the guidelines of each center. Most patients were treated with neoadjuvant chemotherapy, with the addition of hormone therapy in patients with ER expression, and with the addition of trastuzumab in patients with HER2 amplification. In TNBC patients, 21 (78%) of 27 IBC patients and 29 (66%) of 44 non-IBC patients received neoadjuvant chemotherapy. Neoadjuvant regimens based on anthracycline and taxanes, such as T-FAC (weekly paclitaxel followed by 5-fluorouracil [5FU] + doxorubicin + cyclophosphamide), were given at the treating physician’s discretion.

We identified TNBC status with use of GE profiling. Originally, GE data were normalized with the MAS 5 algorithm, mean centered to 600, and log 2 transformed. Because 15 cases had no information on breast cancer-receptor status by immunohistochemistry (IHC) and because we prefer effective utilization for the dataset, we identified ER, PgR, and HER2 receptor status by using, respectively, *ESR1* (probe set 205225_at), *PgR* (208305_at), and *ERBB2* (216836_s_at) mRNA GE data. We constructed receiver operating characteristic curves to measure the predictive accuracy of the logistic regression models, including *ESR1, PgR,* and *ERBB2* mRNA GE levels. Cases with normalized *ESR1* mRNA expression >10.18 were considered ER-positive cases, those with *PgR* mRNA expression >2.907 were considered PR positive, and cases with *ERBB2* mRNA expression >12.54 were considered HER2 amplified [26]. We identified 39 TN-IBC patients. This statistical analysis was performed with BRB-ArrayTools version 3.9.0 α software [27] and R software version 2.7.2 [28].

The World IBC Consortium arrays were not initially quantified as in Lehmann *et al*.; they used the RMA algorithm, and our group used MAS 5.0. For consistent quantification of the arrays, we used the fRMA algorithm, which allows arrays to be analyzed individually or in small batches and then the data to be combined for analysis, to normalize and quantify all the datasets before we applied the Lehmann *et al*. approach. Clinicopathologic characteristics of the TNBC patient cohorts are presented in Table 1.

**Table 1:** **Characteristics of patients with TNBC (*n*** **= 88)**

**Characteristics**	**TN-IBC**			**TN-non IBC**			***P *** **value**
	**No. of patients (%)**			**No. of patients (%)**			
**Total number of patients**	39			49			
**Age (years), median (min-max)**	49 (26-78)			56 (28-77)			0.1681
**Clinical stage of diagnosis**				I	9	(19)	8.904e-09
				II	17	(35)	
	III	27	(69)	III	18	(36)	
	IV	5	(13)	IV	5	(10)	
	Unknown	7	(18)	Unknown	0	(0)	
**Nuclear grade**	I	0	(0)	I	3	(6)	0.2048
	II	4	(10)	II	10	(20)	
	III	33	(85)	III	35	(72)	
	Unknown	2	(5)	Unknown	1	(2)	
**Tumor embolism**	Positive	24	(62)	Positive	3	(6)	3.604e-09
	Negative	12	(30)	Negative	20	(41)	
	Unknown	2	(8)	Unknown	26	(53)	
**Neoadjuvant therapy (stages I-III)**	Positive	21	(78)	Positive	29	(66)	0.1684
	Negative	5	(18)	Negative	15	(34)	
	Unknown	1	(4)	Unknown	0	(0)	

### Identification of TNBC subtypes

In our previous study [25], we reproduced the algorithm of Lehmann *et al*. and extracted the centroids of the seven TNBC subtypes based on the training data (supplementary data). By using that same method, we assigned the 88 TNBC (TN-IBC, *n* = 39; TN-non-IBC, *n* = 49) samples in the World IBC Consortium dataset to a TNBC subtype with the use of both our approximated and Lehmann’s gene signatures. We used the highest Pearson correlation and lowest *P* value as the criteria to determine to which subtype a specific sample belonged. We constructed two 7 × 2 contingency tables based on the gene signatures we obtained and the original ones in Lehmann *et al*. The Fisher Exact test was applied to compare the TN-IBC and TN-non-IBC subtypes.

To determine the power of detecting an extreme shift, we set the margins of the 7 × 2 table of subtypes by IBC status, assigned a 0 to the first cell, randomly allocated the rest, and computed the Fisher test *P* value. We repeated this process 500 times and then moved to the next cell (performing 7,000 tests in all). Our overall power (the chance of getting a Fisher test *P* value of <0.05 when one subtype is extremely different) was 3,304/7,000 = 0.472.

### Survival analysis

We excluded stage IV patients for our survival analysis. Follow-up data were available for 27 TN-IBC and 43 TN-non-IBC patients. The median follow-up periods from diagnosis were 3.39 years (range, 0.84 to 6.48 years) for IBC patients (*n* = 27) and 3.76 years (range, 0.29 to 10.78 years) for non-IBC patients (*n* = 43). We performed log-rank tests to compare clinical outcomes in TN-IBC and TN-non-IBC patients by subtype: overall survival (OS), distant metastasis-free survival (DMFS), and recurrence-free survival (RFS). A Bonferroni-adjusted *P* value (0.05 divided by the number of comparisons made) was used as the cutoff to determine the significance of the log-rank test results regarding the comparisons of TNBC subgroups. Cox models were used to assess the relevance of the subtype classifications overall. We then performed all pairwise comparisons between subgroups with use of log-rank tests with a Bonferroni adjustment to correct for multiple testing.

### GE analysis

We examined differences among TNBC subtypes and between IBC (stage III, IV) and non-IBC (stage III, IV) at the GE level by using feature-by-feature linear mixture models followed by fitting a beta-uniform mixture (BUM) model to control for multiple testing. Numbers of significant genes were counted for some selected false discovery rates (FDRs). The Affymetrix U133 annotation package hgu133a.db was used to export gene symbols for each of the 22,283 probes.

#### Information on microarray data

Data sets for this study have been deposited into the GEO database [29] under accession number GSE45584.

## Results

### Comparison between TN-IBC and TN-non-IBC

We classified each of our IBC and non-IBC samples into one of the seven clusters by using gene signatures and the highest correlation between the sample and the centroid of each cluster. We constructed two 7 × 2 contingency tables based on the gene signatures we obtained (Table 2) and the original ones in Lehmann *et al*. (Table 3). Then we compared the distributions of TN-IBC and TN-non-IBC. With the Fisher Exact test, we found no statistical differences in TNBC-subtype distribution for patients with IBC or patients with non-IBC (*P* = 0.47 and *P* = 0.22, respectively). We could not detect differences between IBC and non-IBC in mRNA expression level that would explain the clinical aggressiveness of IBC. We also analyzed TN-IBC and TN-non-IBC molecular subtypes by using the TNBC population for which triple negativity was identified by both IHC test and mRNA expression level (*N* = 63). We had the same results; no differences in TNBC subtype distribution between IBC and non-IBC were found (see Additional file 2: Table S5A, S5B).

**Table 2:** Distribution of IBC/non-IBC status by TNBC subtype by using MDA gene signatures

	**IBC (%)**	**Non-IBC (%)**	***P *** **value**
Subtype 1 (M)	4 (10.2)	6 (12.2)	0.47
Subtype 2 (IM)	4 (10.2)	12 (24.4)	
Subtype 3 (BL1)	8 (20.5)	7 (14.2)	
Subtype 4 (BL2)	3 (7.6)	2 (4.0)	
Subtype 5 (LAR)	5 (12.8)	5 (10.2)	
Subtype 6 (UNS)	7 (17.9)	12 (24.4)	
Subtype 7 (MSL)	8 (20.5)	5 (10.2)	

**Table 3:** Distribution of IBC/non-IBC status by TNBC subtype by using Lehmann’s gene signatures

	**IBC (%)**	**Non-IBC (%)**	***P *** **value**
M	5 (12.8)	9 (18.3)	0.22
IM	5 (12.8)	12 (24.4)	
BL1	12 (30.7)	10 (20.4)	
BL2	1 (2.5)	4 (8.1)	
LAR	6 (15.3)	6 (12.2)	
UNS	2 (5.1)	5 (10.2)	
MSL	8 (20.5)	3 (6.1)	

Lehmann *et al*. used gene profiles of patients with various stages of disease. However, all IBC cases are stage III or IV. Therefore, we also analyzed stage-matched populations (IBC, *n* = 32; non-IBC, *n* = 23). The results indicated no significant association between TNBC subtypes and IBC status at tumor stage III or above, no matter which set of gene signatures was applied (*P* = 0.47, *P* = 0.77, respectively; data not shown).

### Clinical relevance of TNBC subgroups

**Figure 1:**
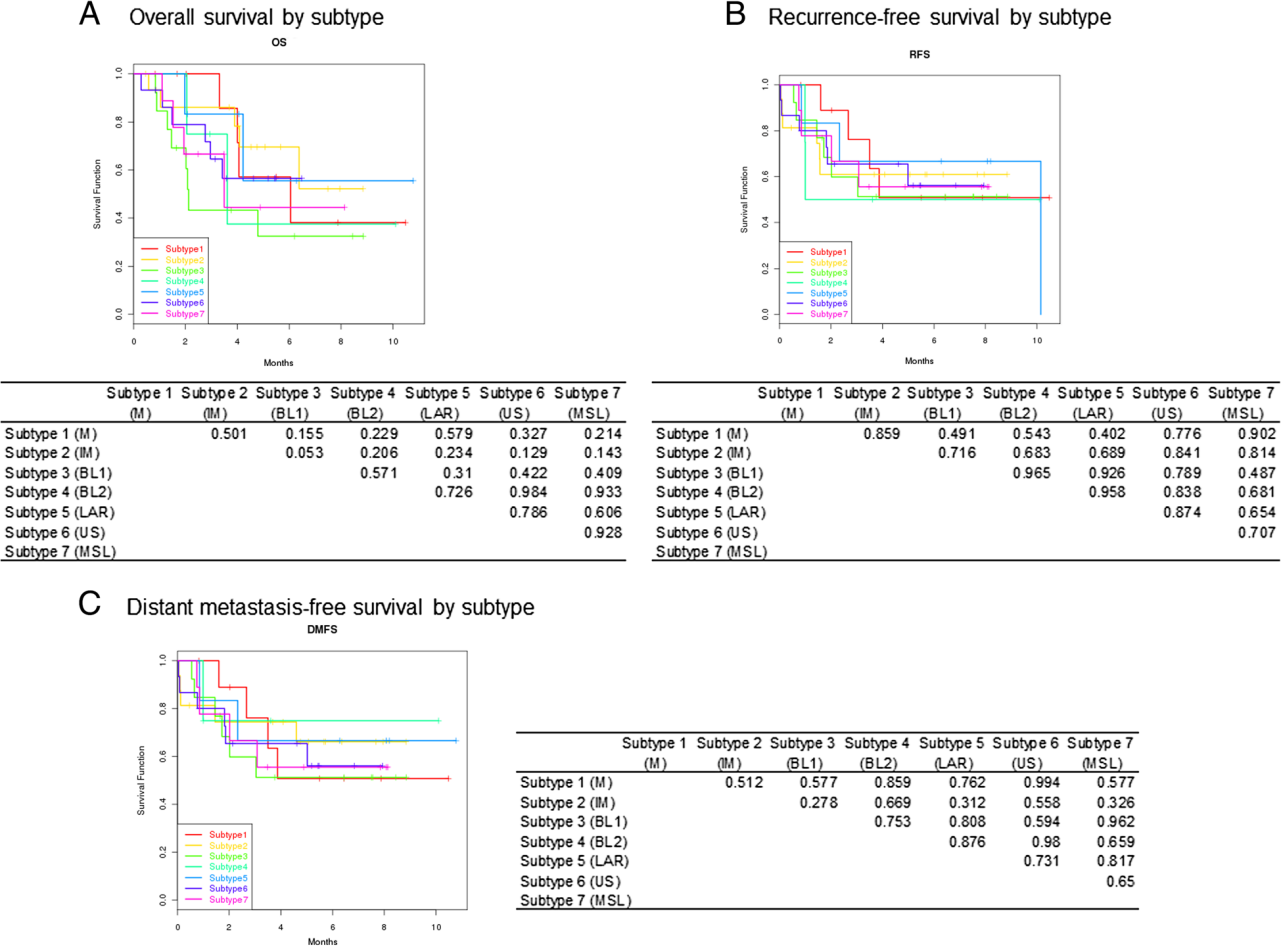
**Kaplan-Meier estimates. (A)** Kaplan-Meier estimates for TNBC subtypes (defined by MDA approximated gene signatures) with respect to overall survival (OS) of patients with stage III disease; **(B)** Kaplan-Meier estimates for TNBC subtypes (defined by MDA approximated gene signatures) with respect to recurrence-free survival (RFS) of patients with stage III disease; **(C)** Kaplan-Meier estimates for TNBC subtypes (defined by MDA approximated gene signatures) with respect to distance metastasis-free survival (DMFS) of patients with stage III disease. The *P* values of the log-rank tests are for each pair of TNBC subtypes with respect to OS. The subtypes were classified by using our approximated gene signatures. The Bonferroni-adjusted *P* value is 0.00238. The *P* values of the log-rank tests are for each pair of TNBC subtypes with respect to RFS. The subtypes were classified by using our approximated gene signatures. The Bonferroni-adjusted *P* value is 0.00238. The *P* values of the log-rank tests are for each pair of TNBC subtypes with respect to DMFS. The subtypes were classified by using our approximated gene signatures. The Bonferroni-adjusted *P* value is 0.00238.

Because the subtypes and distributions of TN-IBC were similar to those of TN-non-IBC, we evaluated the clinical effect of the TN-IBC subgroups among stage III patients, focusing on the prognostic effect for survival data. Log-rank tests and Cox proportional hazards models revealed no significant differences between TNBC subtypes with respect to OS, RFS, and DMFS (Figure 1A through C). We analyzed both our original seven groups and the seven Lehmann *et al*. groups, and results were similar (data not shown).

### Therapeutic relevance of TNBC subgroups

Subsequently, we analyzed GE focusing on predictive effect for therapy selection. Lehmann *et al*. classified breast cancer cell lines according to their subtypes. Xenograft tumors established from their TNBC subtypes displayed differential sensitivity to cisplatin, bicalutamide, and NVP-BEZ235. Lehmann *et al*. showed that their subtypes had a predictive effect relevant to therapy selection. The BL1 and BL2 cell lines, for example, which were associated with the DNA damage response, were highly sensitive to cisplatin; the LAR cell line, which expresses high levels of *AR* mRNA, was highly sensitive to an AR antagonist; and the MSL cell line, associated with the PI3K pathway, was highly sensitive to NVP-BEZ235 (a PI3K/mTOR inhibitor).

**Figure 2:**
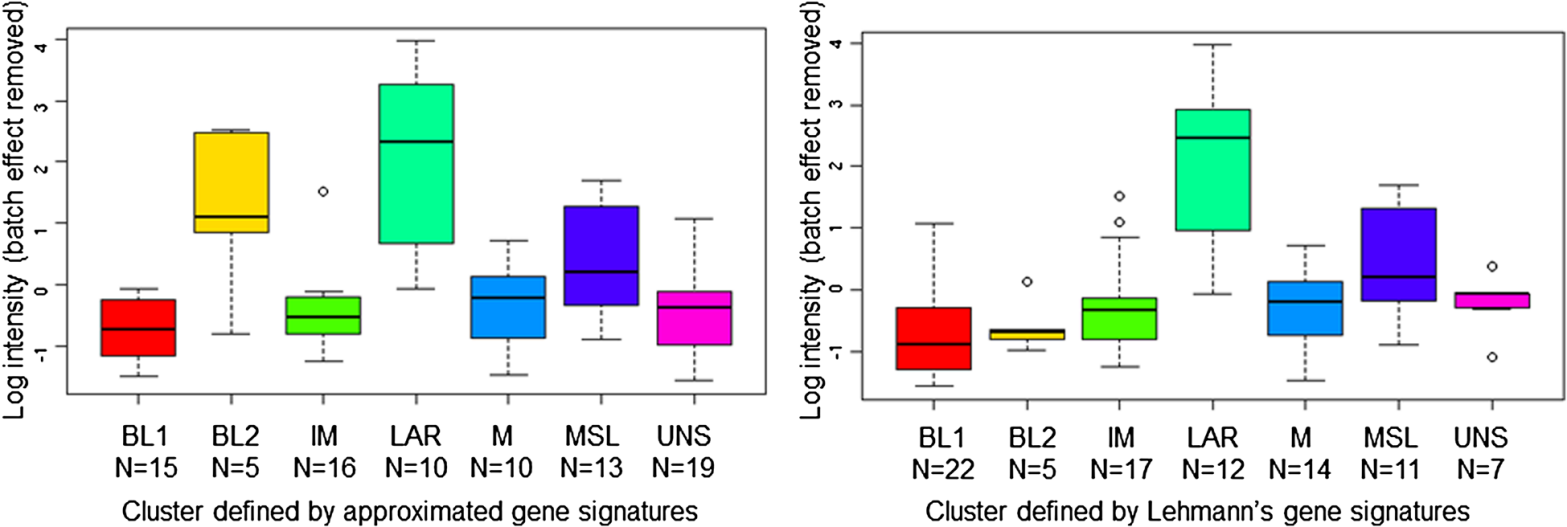
**Boxplots of *AR *** **gene expression levels in TNBC subtypes defined by MDA-approximated gene signatures (left panel) or Lehmann’s gene signatures (right panel).**

On the basis of these results, we analyzed *AR* mRNA expression levels in our subtypes. Only the efficacy of an AR antagonist relative to the expression of AR has been verified; we could not evaluate the efficacy of other drugs through GE levels because cisplatin is not a targeted drug, and the efficacy of NVP-BEZ235 depends not on expression level but on mutations. In our dataset, AR expression was significantly different between TNBC subtypes. The boxplots of GE profiles of AR based on the World IBC Consortium dataset are shown in Figure 2. We selected the probes with the lowest *P* values from linear mixture models to represent these genes. As shown in Figure 2, the AR expression level appears to be higher in subtype 5 defined by our approximated gene signatures and in subtype LAR defined by the gene signatures of Lehmann *et al*. Our approximated TNBC subtype 5 had a high correlation with the LAR subtype. Also, these results revealed the possibility that an AR antagonist could be an effective drug not only for LAR-TN-non-IBC patients but also for LAR-TN-IBC patients. Therefore, both the Lehmann *et al*. study and our study showed that AR antagonists are a reasonable therapy in TNBC and that their efficacy should be tested in prospective studies.

### Comparison of all gene structures in TN-IBC and TN-non-IBC

**Figure 3:**
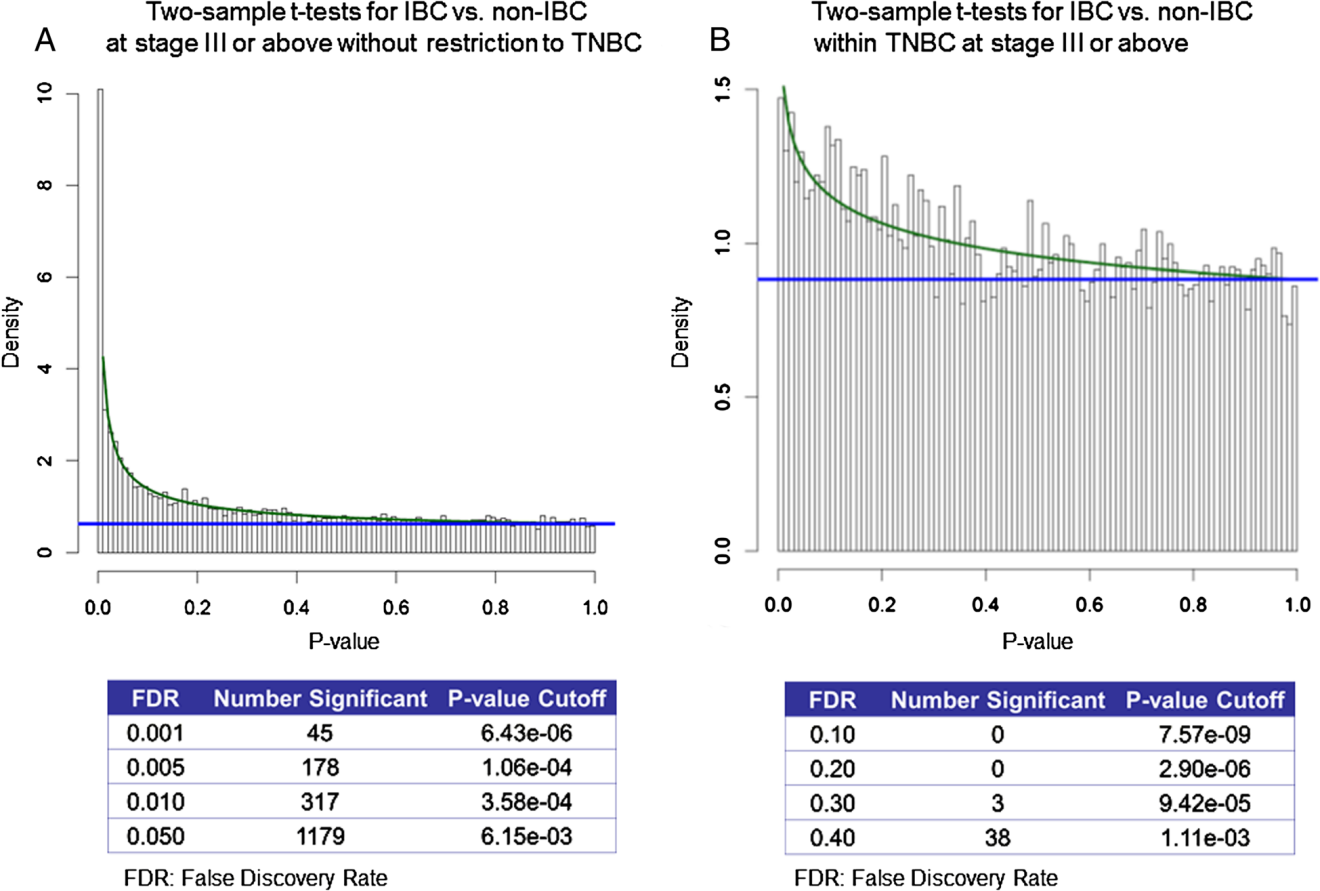
**IBC versus non-IBC at stage III or above. (A)** Those without restriction to TNBC and **(B)** those within TNBC. Histograms of *P* values from two-sample *t* tests for IBC versus non-IBC. The overlaid curves are the fitted BUM models. Counts of significant features with various FDR cutoffs are shown in a comparison of IBC with non-IBC.

To summarize our findings, we did not see a specific biologic profile of TN-IBC. We therefore tested all breast cancer samples (both TNBC and non-TNBC) to compare IBC and non-IBC structures. The results from two-sample *t* tests and the BUM model revealed that at the molecular level, obvious differences existed between IBC and non-IBC for all subtype patients at stage III or above (Figure 3A). At an FDR of 1%, there were 317 significant genes, which means that only three of these genes would be expected to be selected by random chance (false positive). When we restricted our attention to TNBC while still focusing on stage III or above (Figure 3B), and only 38 genes were significant at an FDR of 40%, suggesting no significant difference between IBC and non-IBC restricted to high-tumor-stage TNBC.

## Discussion

Our findings suggest that TN-IBC is a heterogeneous disease. We found seven subtypes, including one unstable subtype, in TN-IBC, and these subtypes showed high correlation with the seven Lehmann *et al*. subtypes. In comparing TN-IBC and TN-non-IBC, the distribution of these seven subtypes showed no statistical differences; the TN-IBC samples were assigned to the seven groups without any predominance, disproving our hypothesis. In addition, we compared all TNBC mRNA gene signatures for IBC and non-IBC and could find no specific molecular signature in the IBC group. Thus, mRNA array analysis showed that IBC and non-IBC have similar biology in TNBC.

Molecular techniques such as DNA microarrays provide novel tools with which to investigate the heterogeneity of breast cancer. To classify subgroups, it is necessary to identify the most appropriate molecular-based therapies. In breast cancer, five molecular subtypes have been repeatedly described in non-IBC [30-32], and these subtypes have been strongly associated with clinical outcome and drug sensitivity. Bertucci *et al*. [9] reported that the expression signatures defining molecular subtypes of non-IBC were also present in IBC, with similar expression patterns. Focusing on the differences between IBC and non-IBC, they suggested that the intrinsic biology associated with cell type was more important to determining the transcriptional pattern than was the clinical aspect of the disease. They further maintained that molecular subtype and inflammatory character are two independent features of breast cancers. Our results are consistent with their interpretation and suggest that to identify the specific biology of IBC, we have to consider not only tumor cells but also their microenvironment and other factors, such as the effects of inflammation, immune pathways, and mutations.

In addition, gene-structure analysis suggested that the specific biology of IBC might be hidden in other breast cancer groups, such as ER-positive and HER2-positive groups. Van Laere *et al*. [33] and Woodward *et al.*[34] also investigated the specific biology of IBC by using the World IBC Consortium dataset but with a different approach. They identified potential genes specific to IBC and found that the differences also were related not to TNBC subtype but to ER-positive subtypes. However, although many studies have examined and compared gene expression between IBC and non-IBC, repeatedly finding clusters associated with receptor subtype, no consistent gene signature associated with IBC has been validated.

It is possible that our dataset was too small to reveal fully differences in distribution, although our 39-case TN-IBC population is the largest so far to be published. As was found previously in the Nguyen *et al.* study [8], we could not investigate the association of each pathway; even if we could classify the TN-IBC subtypes according to those described by Lehmann *et al*., the canonic pathways of each subtype partially overlap those of other subtypes. Moreover, each pathway has correlations with other pathways. Thus, in our study, we could not investigate the associations of several diverse pathways in IBC and therefore could not indicate the differences in TNBC-subtype distribution.

Lehmann *et al*. reported that their subtypes were associated with significant differences in RFS (*P* = 0.0083). Patient RFS was significantly decreased in the LAR subtype compared with the BL1, IM, and MSL subtypes. However, patient characteristics varied, and their therapies and durations of treatment were not standardized. DMFS did not vary significantly between TNBC subtypes. In our cases, in addition to the small numbers, the median follow-up period for IBC patients (*n* = 27) was 3.39 years (range, 0.84 to 6.48 years). We need a longer follow-up time before we can discuss whether these seven subgroups have a prognostic effect. As a result, neither we nor Lehmann *et al.* could verify the effect of TNBC subgroups as prognostic markers.

Lehmann *et al.* showed that their seven TNBC subtypes displayed unique GE and ontologies. They also identified representative subtypes of TNBC cell-line models and predicted that “driver” signaling pathways were pharmacologically targeted in these cell-line models as proof of concept that the analysis of distinct GE signatures can improve therapy selection. In our dataset, we determined that the subgroups that showed the highest *AR* expression level belonged to TN-IBC (subtype 5 and LAR subtype), revealing the possibility that an AR antagonist can be a potentially useful drug for these patients.

## Conclusions

In this study, we addressed the question of whether the aggressive behavior of IBC is tied to TNBC subtype. The results suggested that TN-IBC is a heterogeneous disease. Our findings showed that TNBC subtype classification is not affected by IBC or non-IBC status. TN-IBC and TN-non-IBC had the same subtypes by using mRNA expression profiles. These findings lead to the conclusion that the differences in TN-IBC and TN-non-IBC are from a very subtle molecular difference or have nothing to do with the tumor itself. It is also possible that clinical diagnosis of TN-IBC in particular is not reflected at the molecular level. These results point to a need for elucidating the specific biology of TN-IBC by focusing not only on its direct comparison with non-IBC at the mRNA-expression level but also on its pathway associations; they also show the need for whole-genome deep DNA sequencing (International Cancer Genome Consortium) and for further investigation of microenvironmental differences.

## Abbreviations

BL1: Basal-like 1 subtype; BL2: Basal-like 2 subtype; DMFS: Distant metastasis-free survival; EGFR: Epidermal growth factor receptor; EMT: Epithelial-mesenchymal transition; FDRs: False discovery rates; GE: Gene expression; IBC: Inflammatory breast cancer; IM: Immunomodulatory subtype; LAR: Luminal androgen-receptor subtype; M: Mesenchymal subtype; MSL: Mesenchymal stem-like subtype; OS: Overall survival; RFS: Recurrence-free survival; TN: Triple-negative; TNBC: Triple-negative breast cancer; TN-IBC: Triple-negative inflammatory breast cancer; TN-non-IBC: Triple-negative noninflammatory breast cancer; UNS: Unstable subtype.

## Competing interests

The authors declare that they have no competing interests.

## Authors’ contributions

KAB, YW, HM, and TI carried out the genetic analyses and conducted the statistical analyses. HM, TB, TK, PF, DB, and LD collected and reviewed the clinical information. NTU, SJV, and FB created and organized the World IBC Consortium data. HM and NTU conceived the study and drafted the manuscript. LP, KK, WAW, JMR, WFS, and GNH developed the study design. SK and WFS were involved with tissue-samples collection and interpretation. All authors read and approved the final manuscript.

## Supplementary Material

Additional file 1 **Supplemental information.** Validation of Lehmann *et al*.’s gene expression analysis. Details of sample storage at General Hospital Sint-Augustinus (Translational Cancer Research Unit, Antwerp, Wilrijk, Belgium: 41 IBC and 55 non-IBC). Details of sample storage at Institut Paoli-Calmettes (IPC, Marseille, France: 71 IBC and 139 non-IBC). Details of sample storage at Institut Paoli-Calmettes (IPC, Marseille, France: 71 IBC and 139 non-IBC). Click here for file

Additional file 2 **Supplemental table and figures. Table S1.** The results of ER, PgR, and HER2 status in IHC testing and mRNA expression in the full Consortium population (*N* = 389). **Table S2.** The survival events in each subgroup. **Table S3.** Patient characteristics for the seven TNBC subtypes defined by the Lehmann *et al*. signatures. **Table S4.** Patient characteristics for the seven TNBC subtypes defined by our gene signatures. **Table S5.** The analysis from **Table S2** with TNBC defined by IHC results instead of mRNA expression (*N* = 63). **Figure S1A.** Overall survival by subtype (IHC definition of TNBC). **Figure S1B.** Recurrence-free survival by subtype (IHC definition). **Figure S1C.** Distant metastasis-free survival by subtype (IHC definition). **Figure S2.** The analysis of **Figure S2** with TNBC defined by IHC results instead of mRNA expression (*N* = 63). Click here for file
