# Supplementary material for: Comparison of molecular subtype distribution in triple-negative inflammatory and non-inflammatory breast cancers
Source: Breast Cancer Res. 2013 Nov 25;15(6):R112. doi: 10.1186/bcr3579 (PMC3978878; doi:10.1186/bcr3579)
Supplement: Additional file 1 — Supplemental information. Validation of Lehmann et al.’s gene expression analysis. Details of sample storage at General Hospital Sint-Augustinus (Translational Cancer Research Unit, Antwerp, Wilrijk, Belgium: 41 IBC and 55 non-IBC). Details of sample storage at Institut Paoli-Calmettes (IPC, Marseille, France: 71 IBC and 139 non-IBC). Details of sample storage at Institut Paoli-Calmettes (IPC, Marseille, France: 71 IBC and 139 non-IBC). [file bcr3579-S1.docx]

**Supplemental information**

**Validation of Lehmann et al.’s Gene Expression Analysis**

Reproducing the method of Lehmann and Bauer et al ([3](#_ENREF_3)), we obtained 12 public datasets (323 patients’ DNA microarrays) from their TNBC training set and all 7 public datasets (201 patients’ DNA microarrays) from their TNBC validation set. All of the arrays used were a type of Affymetrix U133 array: U133A, U133 Plus 2.0, or U133AAofAv2. Lehmann et al. used the robust multiarray average (RMA) algorithm to normalize and quantify the data. The data pre-processing and quantification were performed using R statistical software. We used this approach with two modifications. First, we used the frozen RMA (fRMA) algorithm, which allows arrays to be analyzed individually or in small batches and then combines the data for analysis, to normalize and quantify all of the datasets. Second, considering the potential effect of platforms on the gene expression profiles, we converted all of the other platforms to HGU133A by using the “fRMA Tools” R package. We later used linear mixed models to correct for any remaining platform-specific differences. A total of 14,644 probes were selected to represent unique genes.

We performed principal component analysis to check for the existence of batch effects. If batch effects were present, feature-by-feature linear models were applied to remove them. Principal component analysis results revealed an obvious batch effect, which was reduced after the application of linear models.

We applied consensus clustering using 1000 iterations of k-means clustering to assess the classification robustness and to determine the optimal number of clusters. We performed k-means clustering on the genes (n = 1192) with standard deviation >0.8 and used silhouette width (s[i]) to measure the relative closeness of individual samples to their cluster centers. k-means clustering resulted in 6 stable clusters (s[i]>0) with a total of 261 samples and 1 unstable cluster (s[i]<0) containing 62 samples. Our consensus clustering results confirmed that it is reasonable to set k to 7. We labeled these 7 clusters as subtypes 1 to 7. We computed centroids for each cluster from the consensus clustering and then determined the Pearson correlation of each centroid with each sample in the validation set from Lehmann et al. The highest correlation (and lowest *P* value) was used as the criterion with which to determine the subtype that a specific sample belonged to.

The result showed high correlation (P < 2.2×10-16) between our clustering and the original clustering. We also used Cohen’s kappa coefficient to assess the association between our classifications and those reported by Lehmann et al. For the training set, the κ value was 0.662 (n = 277); for the validation set, 0.462 (n = 200). Using large-sample normal approximations, the kappa2 function in the R package gave approximate z-values of 26.1 and 16.2, respectively; the P values were essentially 0. The BL1, M, IM, MSL, and LAR subtypes were more stable than the BL2 and UNS subtypes. We concluded there was high correlation between the results of the Lehmann et al. study and the results we obtained by using approximately the same methods.

**Details of sample storage at General Hospital Sint-Augustinus (Translational Cancer Research Unit, Antwerp, Wilrijk Belgium: 41 IBC and 55 non-IBC)**

Tumor samples were obtained from patients with breast adenocarcinoma treated in the Breast Clinic of General Hospital Sint-Augustinus, Wilrijk, Belgium. Each patient gave written informed consent. All samples were brought to the pathologists and stored in liquid nitrogen within 15 min after excision (median delay, 9 min). The samples were subsequently stored at -180ºC.

The presence of tumor emboli was, as an isolated pathological finding, not sufficient for the diagnosis of IBC. RNA was extracted using the RNeasy Mini Kit (Qiagen, Valencia, CA, USA). RNA samples were hybridized onto Human Genome U133 Plus 2.0 Affymetrix chips in collaboration with the VIB Microarray Facility (O&N, UZ-Gasthuisberg, Leuven, Belgium).

**Details of sample storage at Institut Paoli-Calmettes (IPC, Marseille, France: 71 IBC and 139 non-IBC)**

Pre-treatment tumor tissues were collected from 197 patients with invasive adenocarcinomas who underwent surgical biopsies or initial surgery at Institut Paoli-Calmettes (IPC, Marseille, France) between 1988 and 2008. Each patient gave written informed consent, and the study was approved by the IPC Comité d’Orientation Stratégique. Tumor samples were macrodissected and frozen in liquid nitrogen within 30 min of removal. Before RNA extraction, tumor sections were reviewed by two pathologists (ECJ and JJ) and contained more than 60% of tumor cells. IBC tumors were selected on the basis of meeting clinical criteria for T4d tumor. DNA and RNA were extracted from frozen samples by using guanidium isothiocyanate and a cesium chloride gradient. DNA quality and RNA quality were respectively controlled on polyacrylamide gel electrophoresis and on an Agilent Bioanalyzer (Agilent Technologies, Massy, France).

**Details of sample storage at The University of Texas MD Anderson Cancer Center (United state: 25 IBC and 58 non-IBC)**

At MD Anderson, samples were placed in RNAlater (Ambion, Austin, TX) storage reagent and stored at –80°C until GE analysis.
